# Rifampicin-Monoresistant Tuberculosis Is Not the Same as Multidrug-Resistant Tuberculosis: a Descriptive Study from Khayelitsha, South Africa

**DOI:** 10.1128/AAC.00364-21

**Published:** 2021-10-18

**Authors:** Zubeida Salaam-Dreyer, Elizabeth M. Streicher, Frederick A. Sirgel, Fabrizio Menardo, Sonia Borrell, Miriam Reinhard, Anna Doetsch, Patrick G. T. Cudahy, Erika Mohr-Holland, Johnny Daniels, Anzaan Dippenaar, Mark P. Nicol, Sebastien Gagneux, Robin M. Warren, Helen Cox

**Affiliations:** a Division of Medical Microbiology, Department of Pathology, University of Cape Town, Cape Town, South Africa; b DST/NRF Centre of Excellence for Biomedical Tuberculosis Research/SAMRC Centre for Tuberculosis Research, Division of Molecular Biology and Human Genetics, Faculty of Medicine and Health Sciences, Stellenbosch University, Stellenbosch, South Africa; c Swiss Tropical and Public Health Institute, Basel, Switzerland; d University of Basel, Basel, Switzerland; e Section of Infectious Diseases, Department of Internal Medicine, Yale School of Medicine, New Haven, Connecticut, USA; f Médecins Sans Frontières, Khayelitsha, Cape Town, South Africa; g Tuberculosis Omics Research Consortium, Family Medicine and Population Health, Institute of Global Health, Faculty of Medicine and Health Sciences, University of Antwerp, Antwerp, Belgium; h Division of Infection and Immunity, School of Biomedical Sciences, University of Western Australia, Perth, Australia; i Institute of Infectious Disease and Molecular Medicine and Wellcome Centre for Infectious Disease Research, University of Cape Town, Cape Town, South Africa

**Keywords:** tuberculosis, drug resistance, whole-genome sequencing, rifampin-monoresistant TB, multidrug-resistant TB, MDR-TB, drug resistance evolution, human immunodeficiency virus, rifampin

## Abstract

Rifampin monoresistance (RMR; rifampin resistance and isoniazid susceptibility) accounts for 38% of all rifampin-resistant tuberculosis (RR-TB) in South Africa and is increasing. We aimed to compare RMR-TB with multidrug-resistant TB (MDR-TB) in a setting with high TB, RR-TB, and HIV burdens. Patient-level clinical data and stored RR Mycobacterium tuberculosis isolates from 2008 to 2017 with available whole-genome sequencing (WGS) data were used to describe risk factors associated with RMR-TB and to compare RR-conferring mutations between RMR-TB and MDR-TB. A subset of isolates with particular RR-conferring mutations were subjected to semiquantitative rifampin phenotypic drug susceptibility testing. Among 2,041 routinely diagnosed RR-TB patients, 463 (22.7%) had RMR-TB. HIV-positive individuals (adjusted odds ratio [aOR], 1.4; 95% confidence interval [CI], 1.1 to 1.9) and diagnosis between 2013 and 2017 versus between 2008 and 2012 (aOR, 1.3; 95% CI, 1.1 to 1.7) were associated with RMR-TB. Among 1,119 (54.8%) patients with available WGS data showing RR-TB, significant differences in the distribution of *rpoB* RR-conferring mutations between RMR and MDR isolates were observed. Mutations associated with high-level RR were more commonly found among MDR isolates (811/889 [90.2%] versus 162/230 [70.4%] among RMR isolates; *P* < 0.0001). In particular, the *rpoB* L430P mutation, conferring low-level RR, was identified in 32/230 (13.9%) RMR isolates versus 10/889 (1.1%) in MDR isolates (*P* < 0.0001). Among 10 isolates with an *rpoB* L430P mutation, 7 were phenotypically susceptible using the critical concentration of 0.5 μg/ml (range, 0.125 to 1 μg/ml). The majority (215/230 [93.5%]) of RMR isolates showed susceptibility to all other TB drugs, highlighting the potential benefits of WGS for simplified treatment. These data suggest that the evolution of RMR-TB differs from MDR-TB with a potential contribution from HIV infection.

## INTRODUCTION

Globally, an estimated 465,000 individuals became ill with rifampin-resistant tuberculosis (RR-TB) in 2019 (1). Among these, 78% were estimated to have multidrug-resistant tuberculosis (MDR-TB) with resistance to both rifampin (RIF) and isoniazid (INH), while the remainder had rifampin monoresistant TB (RMR-TB, defined as RIF resistance and INH susceptibility). While RMR-TB represents 22% of all RR-TB globally, this percentage varies widely across countries with high RR-TB burdens, ranging from <1% in several countries to more than 40% in countries as diverse as Kenya and Tajikistan (1). In South Africa, RMR-TB constitutes 38% of the more than 13,000 RR-TB cases diagnosed annually (1). In addition, national TB drug resistance surveys have suggested that RMR-TB increased significantly between 2002 and 2012 in South Africa, while the proportion of all TB cases with MDR-TB remained relatively constant (2).

RIF resistance in Mycobacterium tuberculosis is caused by mutations predominantly in the rifampin resistance-determining region (RRDR) of the RNA polymerase β subunit (*rpoB*) gene (3). While any nonsynonymous mutation in the RRDR is considered to confer RR, there is now increasing evidence that some *rpoB* mutations, often described as “disputed” or “discordant,” are associated with decreased RIF susceptibility. The elevated MICs caused by these mutations show a range of values around both the epidemiological cutoff value and the critical concentration (CC) (4, 5). Associations between these low-level-RIF-resistance variants and poor patient outcomes (5–8) have led to a recent change in the CC value recommended by the World Health Organization (WHO) for RIF from 1.0 to 0.5 μg/ml in MGIT 960 and Middlebrook 7H10 media to encompass low-level resistance (9).

Despite the large RMR-TB burden globally, little is known about the emergence and evolution of RMR-TB compared to MDR-TB. In addition, while the prevalence of discordant or low-level *rpoB* variants likely varies by setting (10–12), association with varying prevalence of RMR-TB is unknown. Given the high and increasing prevalence of RMR-TB in South Africa, we aimed to describe RMR-TB in detail in Khayelitsha, a periurban district in Cape Town, South Africa. This included risk factors for RMR-TB, the distribution of RR-conferring mutations determined through whole-genome sequencing (WGS), and RIF MICs among a subset of isolates displaying *rpoB* mutations described as conferring low-level RIF resistance.

## RESULTS

### RR-TB cohort.

**FIG 1:**
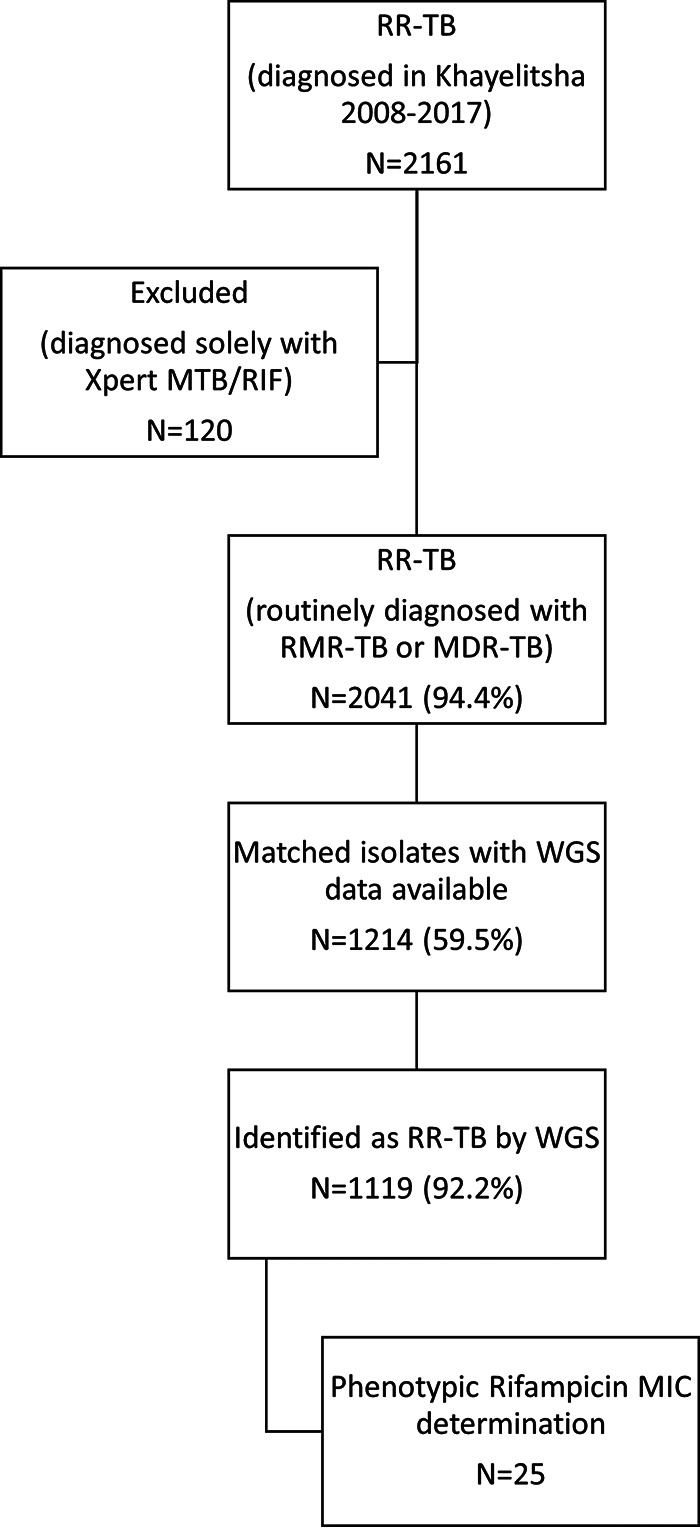
Schematic showing cohort size, availability of whole-genome sequencing data, and subset with MIC determination.

Between 2008 and 2017 inclusive, 2,161 individuals were diagnosed with bacteriologically confirmed RR-TB in Khayelitsha. Among these, 120 (5.6%) were excluded from the cohort as they were diagnosed with RR-TB solely on the basis of an Xpert MTB/RIF or Xpert Ultra test result, without further drug susceptibility testing (DST) to confirm RR or diagnose INH resistance. Valid WGS sequencing data were available for 1,207/2,041 (59.1%) patients; however, RR was identified by TB Profiler in 1,119/1,207 (92.7%) isolates, and among these, 25 underwent RIF MIC determination (Fig. 1).

### Routine RMR-TB diagnosis.

Overall, 463/2,041 (22.7%) individuals were diagnosed with RMR-TB. On univariate analysis, HIV-positive individuals were more likely to have RMR-TB than MDR-TB compared to those who were HIV negative (Table 1). RMR-TB also made up a greater proportion of all RR-TB in the second half of the study decade. On multivariate analysis, HIV positivity, age between 35 and 44 years, and diagnosis in the second half of the study period were significantly associated with RMR-TB compared to MDR-TB (Table 1).

**TABLE 1:** Association between demographic and clinical factors and routinely diagnosed RMR-TB among RR-TB patients in Khayelitsha between 2008 and 2017 inclusive

	No. (%) in group		Odds ratio (95% confidence interval)^*a*^	
Characteristic	Total (*n* = 2,041)	RMR-TB (*n* = 463)	Univariate	Multivariable
Sex				
Female	991	223 (22.5)	0.98 (0.80–1.21)	0.90 (0.73–1.12)
Male	1,050	240 (22.9)	1.0	1.0
Age (yrs)				
0–24	319	76 (23.8)	1.0	1.0
25–34	744	184 (24.7)	1.05 (0.77–1.43)	0.91 (0.66–1.26)
35–44	634	131 (20.7)	0.83 (0.60–1.15)	**0.68 (0.48–0.97)**
45+	344	72 (20.9)	0.85 (0.59–1.22)	0.73 (0.50–1.07)
HIV status				
Negative	503	95 (18.9)	1.0	1.0
Positive	1,490	354 (23.8)	**1.34 (1.04–1.72)**	**1.43 (1.08–1.89)**
Unknown	48	14 (29.2)	1.77 (0.91–3.43)	**2.51 (1.23–5.10)**
Previous TB treatment				
No	622	135 (21.7)	1.0	1.0
Yes	1,349	316 (23.4)	1.11 (0.88–1.39)	1.13 (0.90–1.43)
Unknown	70	12 (17.1)	0.75 (0.39–1.43)	0.62 (0.31–1.24)
Yr diagnosed				
2008–2012	1,066	219 (20.5)	1.0	1.0
2013–2017	975	244 (25.0)	**1.29 (1.04–1.59)**	**1.34 (1.09–1.66)**

a Boldface indicates statistical significance.

### Detection of rifampin and other TB drug resistance using whole-genome sequencing.

WGS data were significantly more likely to be available from patients who were HIV positive and those who initiated RR-TB treatment, although these differences were small overall (Table 2).

**TABLE 2:** Comparison between patients with available TB isolate WGS data and those without

Characteristic	No. (%) with:		
	WGS not available	WGS available	*P* value^*a*^
Total no.	827	1214	
Female	416 (50.3)	575 (47.4)	0.21
Median age (IQR^*b*^)	34 (27-41)	34 (28-41)	0.70
HIV positive (% of known)	625 (75.6)	865 (71.3)	0.0095
Previous TB treatment	535 (64.7)	814 (67.1)	0.77
Yr diagnosed			
2008–2012	423 (39.7)	643 (60.3)	0.44
2013–2017	404 (41.4)	571 (58.6)	
RMR-TB (routine diagnosis)	202 (24.5)	261 (21.5)	0.13
Initiated RR-TB treatment	679 (82.1)	1107 (91.2)	<0.0001

a Chi-squared for difference in proportions.

b IQR, interquartile range.

Among the 1,119 isolates where mutations known to confer any level of RR were found, 230 (20.6%) were identified as RMR and 899 (79.4%) were MDR. There were clear differences in the distribution of RR-conferring mutations between RMR and MDR isolates (Table 3). Notably, the common high-confidence *rpoB* S450L mutation was identified in only 73/230 (31.7%) RMR isolates compared to 625/889 (70.3%) MDR isolates (*P* < 0.0001). In contrast, the *rpoB* L430P mutation, previously described as conferring low-level RR, was identified in 32/230 (13.9%) RMR isolates, compared to only 10/889 (1.1%) MDR isolates (*P* < 0.0001). Overall, high-confidence RR-conferring mutations were identified in 162/230 (70.4%) of RMR isolates, compared to 811/889 (90.2%) of MDR isolates (*P* < 0.0001).

**TABLE 3:** Comparison of *rpoB* mutations between RMR and MDR isolates and description of the confidence level for specific RR-conferring mutations^*a*^

Confidence classification and *rpoB* RR-conferring mutation(s)	No. (%) of isolates		*P* value^*b*^
	RMR (*n* = 230)	MDR (*n* = 889)	
High			
S450L	73 (31.7)	625 (70.3)	<0.0001
D435V	2 (0.9)	76 (8.5)	<0.0001
H445Y	37 (16.1)	25 (2.8)	<0.0001
H445D	18 (7.8)	28 (3.1)	0.0015
H445L	9	10	
D435F	12	1	
H445R	3	3	
S450F	0	6	
T400A, S450L	0	6	
S450W	1	4	
S450W, H445N	0	5	
Q432P	0	4	
Q432L	0	3	
Q432K	0	3	
S431G, D435G	0	3	
D435G, L430P	0	2	
H445Y, D435Y	1	1	
I452P, H445D	2	0	
D435A	1	0	
D435G	1	0	
D435V, L430P	1	0	
D435V, L452P	0	1	
D435V, S450L	0	1	
H445G	0	1	
I491F, S450L	1	0	
S431T, L430P	0	1	
S450Y	0	1	
V170F, S450L	0	1	
Total	162 (70.4)	811 (90.2)	<0.0001

Moderate			
L452P	16 (7.0)	28 (3.2)	0.014
D435Y	7 (3.0)	30 (3.4)	0.83
S441L	6	0	
D435Y, S428T	0	1	
L430R, D435Y	0	1	
L452P, L430P	1	0	
M434I, D435Y	0	1	
P454H, D435Y	0	1	
Total	30 (13.0)	62 (7.0)	0.0046

Minimal			
L430P	32 (13.9)	10 (1.1)	<0.0001
H445N	3	2	
I491F	0	1	
Total	35 (15.2)	13 (1.5)	<0.0001

Unclassified			
Del1306	2	0	
Del1295	0	1	
Del1302	0	1	
R448K	0	1	
T427A	1	0	
Total	3	3	

a Where >1 mutation was identified, the highest confidence mutation was specified.

b Chi-squared for difference in proportions.

The presence of additional TB drug resistance was also strikingly different between RMR and MDR isolates. Only 15/230 (6.5%) RMR isolates displayed additional drug resistance-conferring mutations. This contrasts with MDR isolates, where 815/899 (90.7%) showed other resistance-conferring mutations, in addition to those conferring RIF and INH resistance (Table 4).

**TABLE 4:** Complete drug resistance profile based on WGS (TB Profiler) among isolates identified with RR (MDR and RMR)

TB type and drug resistance profile^*a*^	No. (%) of isolates
MDR	
HRZE, ETH	171 (19.2)
HR, ETH	135 (15.2)
HR	84 (9.4)
HRE, ETH	72 (8.1)
HRE	63 (7.1)
HRZE, FLQ, ETH	63 (7.1)
HRZ, ETH	61 (6.9)
HRZE, FLQ, INJ, ETH	54 (6.1)
HRZE, INJ, ETH	46 (5.2)
HRZE	42 (4.7)
HRZE, FLQ, INJ, ETH, CYC	17 (1.9)
HRZ	13 (1.5)
HRZE, INJ, ETH, CYC	9 (1.0)
HRE, FLQ, ETH	8 (0.9)
HRE, FLQ, INJ, ETH	7 (0.8)
HRZE, FLQ, ETH, CYC	7 (0.8)
HRZ, FLQ, ETH	6 (0.7)
HRZE, ETH, CYC	5 (0.6)
HRZ, PAS	4 (0.4)
HRZE, FLQ, INJ	4 (0.4)
HRZ, INJ, ETH	3 (0.3)
HRZE, FLQ	3 (0.3)
HRE, FLQ	2 (0.2)
HRE, INJ, ETH	2 (0.2)
HRZE, FLQ, ETH, PAS	2 (0.2)
HR, DEL	1 (0.1)
HR, FLQ, ETH	1 (0.1)
HRE, INJ	1 (0.1)
HRZ, FLQ, INJ, ETH	1 (0.1)
HRZE, FLQ, INJ, ETH, PAS	1 (0.1)
HRZE, PAS	1 (0.1)
Total	889

RMR	
R	215 (93.5)
R, ETH	4 (1.7)
R, INJ	3 (1.3)
RZ	3 (1.3)
RE	2 (0.9)
R, FLQ	1 (0.4)
RE, ETH	1 (0.4)
RZE	1 (0.4)

a Abbreviations: H, isoniazid; R, rifampin; Z, pyrazinamide; E, ethambutol; ETH, ethionamide; FLQ, fluoroquinolone; INJ, second-line injectables; CYC, cycloserine; PAS, *para*-aminosalicylic acid; DEL, delamanid.

### Associations with particular *rpoB* mutations.

Given the different *rpoB* mutation distributions, we assessed factors associated with the S450L mutation, conferring high-level RR, and L430P, associated with low-level RR. On multivariate analysis, only MDR-TB was significantly associated with the S450L *rpoB* mutation. Similar results were seen for associations with any high-confidence *rpoB* mutation (data not shown). In contrast, RMR-TB, being female, and having no previous TB treatment were associated with the *rpoB* L430P mutation (Table 5). HIV infection was not associated with either mutation on multivariate analysis.

**TABLE 5:** Multivariate logistic regression analysis of factors potentially associated with either the S450L or L430P *rpoB* mutation

Characteristic	Multivariate OR (95% confidence interval) for *rpoB* mutation^*a*^	
	S450L	L430P
Sex		
Female	1.09 (0.83–1.42)	**0.46 (0.23–0.95)**
Male	1.0	1.0
Age (yrs)		
0–24	1.0	1.0
25–34	1.14 (0.76–1.70)	0.61 (0.22–1.65)
35–44	1.03 (0.67–1.57)	1.53 (0.57–4.08)
45+	1.26 (0.80–2.01)	0.57 (0.17–1.91)
Drug resistance profile		
MDR	**5.03 (3.66–6.85)**	1.0
RMR	1.0	**12.84 (6.33–26.03)**
HIV status		
Negative	1.0	1.0
Positive	0.88 (0.64–1.22)	0.70 (0.32–1.52)
Unknown	1.37 (0.42–4.43)	3.06 (0.32–29.01)
Previous TB treatment		
No	1.0	1.0
Yes	1.06 (0.80–1.42)	**0.40 (0.20–0.79)**
Unknown	1.44 (0.50–4.12)	
Yr diagnosed		
2008–2012	1.0	1.0
2013–2017	0.82 (0.63–1.06)	1.16 (0.60–2.26)

a Boldface indicates statistical significance.

### Phenotypic rifampin resistance and *rpoB* mutations.

Quantitative phenotypic MIC testing was performed for 25 RR isolates selected based on WGS data showing the most common minimal-confidence (*n* = 13) or moderate-confidence (*n* = 12) RR-conferring mutations. Overall, 15/25 (60%) were determined to be phenotypically resistant to RIF when 0.5 μg/ml was used as the CC. Among the 10 isolates with the *rpoB* L430P mutation, MICs ranged from 0.125 μg/ml to 1 μg/ml, with 7 isolates (70%) determined to be phenotypically RIF susceptible (Table 6). Notably, all patients from whom these isolates were derived were routinely diagnosed as having RR-TB with either Xpert and/or LPA.

**TABLE 6:** Quantitative phenotypic DST for rifampin by *rpoB* mutation among 25 RR isolates

*rpoB* mutation	Confidence level	WGS DR profile	Rifampin MIC (μg/ml)	No. of isolates
L430P	Minimal	RMR	0.125^*a*^	4
L430P	Minimal	RMR	0.25^*a*^	2
L430P	Minimal	RMR	0.5^*a*^	1
L430P	Minimal	RMR	1^*a*^	1
L430P	Minimal	MDR	1^*a*^	2
H445N	Minimal	MDR	20	2
I491F	Minimal	MDR	1^*a*^	1
S441L	Moderate	RMR	10	2
D435Y	Moderate	RMR	1^*a*^	2
D435Y	Moderate	MDR	2	2
L452P	Moderate	RMR	0.5^*a*^	2
L452P	Moderate	MDR	2	3
L452P	Moderate	MDR	10	1

a Phenotypically rifampin susceptible based on critical concentration of 1.0 μg/ml.

## DISCUSSION

RMR-TB forms a significant proportion of the total RR-TB burden in this high-TB, -RR-TB, and -HIV setting. Overall, 23% of all routinely diagnosed RR-TB patients were diagnosed with rifampin-resistant but isoniazid-susceptible TB, which we have defined as RMR-TB. This figure is slightly lower than the estimate of 29% for the Western Cape Province of South Africa and lower than the 38% reported for South Africa overall (1, 2). There was, however, a significant increase in the proportion of RMR-TB among all RR-TB cases in the second half of the decade included in this study, consistent with that observed across South Africa (2).

In this large cohort, there were significant differences in the distribution of RR-conferring mutations between RMR and MDR isolates. High-confidence RR-conferring mutations were more commonly found among MDR isolates than RMR isolates; only 70% of RMR isolates were found to have mutations described as high confidence in conferring RIF resistance. This is similar to recent data from New York, where RMR-TB was also associated with low-confidence *rpoB* mutations and low-level phenotypic RR (13). In particular, in our setting, the most common *rpoB* S450L mutation was identified in a much higher proportion of MDR isolates than RMR isolates, while the rarer or disputed *rpoB* L430P mutation, with minimal or low-level confidence in conferring RR, was found in 14% of RMR isolates compared to only 1% of MDR isolates. While the *rpoB* L430P mutation has previously been described in various settings (11, 12, 14), it has not been reported to be associated with RMR-TB. When semiquantitative phenotypic DST was performed on 10 isolates with the L430P mutation, the majority were RIF susceptible at the revised critical concentration of 0.5 μg/ml, suggesting that a single breakpoint for defining resistance may not be sufficient to identify low-level resistance that may well still be clinically significant (5, 6).

RMR-TB was also significantly associated with HIV positivity, a finding also shown in other studies (15–18). However, there have been few representative cohort studies assessing this association in high-HIV-burden and high-TB-burden settings. There are several mechanisms potentially underlining any association between HIV and RMR-TB. First, RMR isolates may be less fit than their MDR counterparts, thereby leading to a greater risk of infection and disease among immunocompromised HIV-positive individuals compared to HIV-negative individuals. A recent multicenter study found that RR isolates from HIV-positive patients were more likely to carry *rpoB* mutations associated with fitness costs, although there were insufficient RMR-TB cases to confirm a specific association (19). While the higher proportion of the *rpoB* S450L mutation, which is associated with a low or no fitness cost (20), among MDR isolates in our data supports this, we did not demonstrate an independent association between HIV and the presence (or absence) of the *rpoB* S450L mutation. HIV was also not an independent predictor of the *rpoB* L430P mutation, which has been associated with delayed growth in culture, suggestive of lower bacterial fitness (21). Second, HIV could be associated with the emergence of RR- and RMR-TB through an increased risk of resistance acquisition during TB treatment. A particular association between HIV infection and the acquisition of RR during TB treatment, predominantly among severely immunocompromised patients, has been shown (22–24). This may be attributed to altered pharmacokinetics, potentially associated with drug malabsorption (25). However, while HIV-positive individuals were 40% more likely to have RMR-TB in our study, there was no independent association between RMR-TB and previous TB treatment.

In addition to the different *rpoB* mutation profile seen between RMR and MDR isolates, there were substantially different patterns of resistance to TB drugs other than RIF and INH. Most RMR isolates were resistant only to RIF, with less than 3% of isolates being resistant to other first-line TB drugs. These data suggest that RMR-TB treatment regimens could be tailored to include first-line TB drugs to which the isolate remains susceptible, potentially by using increased RIF doses or treatment with other rifamycins to overcome low-level RIF resistance (26–28).

Currently, all RR-TB patients, including those with RMR-TB, are treated with predominantly second-line TB regimens, with the addition of INH in some instances (29). This recommendation has been reiterated by the recent WHO technical expert review group (9). While recommended second-line RR-TB regimens have improved in recent years, they remain lengthy and poorly tolerated by patients (30). These data also highlight the potential benefits of using whole-genome or targeted-genome sequencing to individualize RR-TB treatment, particularly for RMR-TB patients, although the wide range of MICs demonstrated here suggests that associations between the presence of specific mutations and phenotypic resistance are not always clear (31, 32).

While there were significant differences between RR-TB patients for whom WGS data were available and those for whom they were not, these were small in magnitude and therefore unlikely to have had a major impact on the striking differences seen between RMR-TB and MDR isolates in this data set. Missing sequencing data were predominantly due to lack of availability of stored isolates in the biobank, in turn likely due to logistical challenges in capturing all TB isolates that are routinely identified as RR over such a long period. In addition, only a small subset of isolates showing *rpoB* mutations described as having minimal or moderate confidence in conferring RR underwent phenotypic MIC determination. Enlarging this subset would provide more data on the seemingly wide variability in MICs among isolates with the same mutation. MICs were also determined only in liquid media, whereas the solid-agar proportion method might have been more sensitive in detecting low-level RIF resistance (33). Finally, as this was a retrospective cohort, we did not have pharmacokinetic data available.

This large-cohort study describing a representative community sample of RR-TB patients shows significant differences between RMR-TB and MDR isolates in terms of RR-conferring *rpoB* mutations and TB drug resistance profiles. While HIV was associated with RMR-TB overall, HIV-positivity did not appear to be related to the observed differences in *rpoB* mutation distribution. Further work on this and other cohorts is required to assess the relative contributions of transmission and resistance acquisition to both RMR-TB and MDR-TB, and particularly the potential role of HIV in the increase in RMR-TB over time.

## MATERIALS AND METHODS

This retrospective, cross-sectional study received ethical approval from both the University of Cape Town (UCT HREC 416/2014) and Stellenbosch University (SU N09/11/296). Patient consent for storage and sequencing of TB isolates was waived.

### Study setting and routine RR-TB diagnosis.

Khayelitsha has an estimated population of 450,000 individuals with high levels of unemployment and poverty. The annual RR-TB case notification rate is estimated at 55/100,000/year, and approximately 70% of RR-TB patients are HIV positive (34). Since 2008, most RR-TB patients are managed as outpatients, with clinical, demographic, and routine laboratory data collected routinely as previously described (34).

In late 2011, Xpert MTB/RIF was introduced for routine diagnosis of TB, including detection of RR among all individuals with presumptive TB; prior to this, only high-risk individuals, such as those with previous TB treatment, were tested for RR-TB. Mycobacterial culture is routinely done on samples from HIV-positive patients with presumptive TB, in whom Xpert MTB/RIF is negative for TB diagnosis, and on samples from patients with RR-TB. Line probe assay (LPA) testing is subsequently done to confirm RR and determine INH resistance on all RR isolates. Once RR is diagnosed, either with Xpert MTB/RIF (or more recently Xpert MTB/RIF Ultra) or with LPA, second-line TB drug resistance testing is done. Specimens from patients with RR-TB but INH susceptibility on LPA testing are further tested for phenotypic INH resistance at a CC of 0.1 μg/ml.

### Whole-genome sequencing.

Individual, patient-level clinical data from RR-TB patients diagnosed between 2008 and 2017 were linked to RR isolates routinely stored at −80°C in a biobank. Matched, stored isolates closest to the date of first RR-TB diagnosis were subcultured into M. tuberculosis Bactec mycobacterial growth indicator tubes (MGITs) for subsequent DNA extraction and quantitative phenotypic DST.

Genomic DNA was extracted using the phenol-chloroform method as previously described (35). DNA concentrations were measured using a Nanodrop ND-1000 spectrophotometer, and DNA integrity was checked by agarose gel electrophoresis (1% gel). WGS was performed on libraries prepared from purified genomic DNA using Illumina Nextera XT library and NEBNext Ultra TM II FS DNA library preparation kits. Sequencing was performed using the Illumina HiSeq 2500 or NextSeq 500 platform. WGS-based drug resistance profiles and RR-conferring mutations were determined using TB Profiler (command line; version 2.8.12) (36). WGS data were excluded if the mean read depth across drug resistance-conferring sites was <20. The M. tuberculosis numbering system was used to describe *rpoB* mutations (37).

### Semiquantitative phenotypic drug susceptibility testing.

Based on WGS data, a convenience subsample of RR isolates (including MDR-TB and RMR-TB) identified with a range of common minimal- or moderate-confidence RR-conferring mutations (38) were tested for MIC determination. RIF MICs were determined using the Bactec MGIT 960 system in order to describe how close MICs might be to the specified critical concentration. Testing was as recommended by the manufacturer (Bactec MGIT; Becton Dickinson, MD, USA) at doubling drug concentrations ranging from 0.03 to 1.0 μg/ml, including 2.0, 6.0, 10, and 20 μg/ml. A fully susceptible M. tuberculosis H37Rv strain (ATCC 27294) was used for quality assurance purposes to confirm the precision of each batch of reagents and drugs.

### Data analysis.

For the entire RR-TB cohort, drug resistance profile was defined based on routine diagnostic testing; RMR-TB was defined as RIF resistance and INH susceptibility regardless of other TB drug resistance, while MDR-TB was defined as resistance to both RIF and INH, again regardless of other TB drug resistance, including second-line TB drug resistance. For the WGS cohort, we defined RR-TB as any *rpoB* mutation identified by TB Profiler as conferring rifampin resistance. This included *rpoB* mutations associated with low-level RR. RMR-TB and MDR-TB were defined in the WGS cohort similarly to the entire cohort. RR-conferring mutations were classified as minimal, moderate, and high confidence with regard to conferring RR, as previously described (38). Previous TB treatment was defined for a patient who had received ≥1 month of anti-TB drugs in the past. Chi-squared analyses (2-sided) were used to compare proportions, and multivariate logistic regression analyses were used to assess variables associated with RMR-TB and the presence of low-level RR-conferring *rpoB* mutations. Variables were entered into multivariate models based on univariate significance or potential relevance based on literature. Data were analyzed with SPSS (IBM Statistics, version 26).

### Data availability.

The bacterial DNA sequencing data are available at the European Nucleotide Archive. The accession number is PRJEB45389.
